# Insights into Cystic Fibrosis Polymicrobial Consortia: The Role of Species Interactions in Biofilm Development, Phenotype, and Response to In-Use Antibiotics

**DOI:** 10.3389/fmicb.2016.02146

**Published:** 2017-01-13

**Authors:** Andreia P. Magalhães, Susana P. Lopes, Maria O. Pereira

**Affiliations:** Centre of Biological Engineering (CEB), Laboratório de Investigação em Biofilmes Rosário Oliveira (LIBRO), University of Minho, BragaPortugal

**Keywords:** polymicrobial interaction, cystic fibrosis, antibiotic therapy, *Pseudomonas aeruginosa*, *Staphylococcus aureus*, *Inquilinus limosus*, Stenotrophomonas maltophilia

## Abstract

Cystic Fibrosis (CF) airways disease involves complex polymicrobial infections where different bacterial species can interact and influence each other and/or even interfere with the whole community. To gain insights into the role that interactions between *Pseudomonas aeruginosa* in co-culture with *Staphylococcus aureus, Inquilinus limosus*, and *Stenotrophomonas maltophilia* may play in infection, the reciprocal effect during biofilm formation and the response of dual biofilms toward ciprofloxacin under *in vitro* atmospheres with different oxygen availabilities were evaluated. Biofilm formation kinetics showed that the growth of *S. aureus, I. limosus*, and *S. maltophilia* was disturbed in the presence of *P. aeruginosa*, under both aerobic and anaerobic environments. On the other hand, under aerobic conditions, *I. limosus* led to a decrease in biofilm mass production by *P. aeruginosa*, although biofilm-cells viability remains unaltered. The interaction between *S. maltophilia* and *P. aeruginosa* positively influenced dual biofilm development by increasing its biomass. Compared with monocultures, biomass of *P. aeruginosa*+ *S. aureus* biofilms was significantly reduced by reciprocal interference. When grown in dual biofilms with *P. aeruginosa*, ciprofloxacin was less effective against *S. aureus, I. limosus, and S. maltophilia*, with increasing antibiotic doses leading to drastic inhibitions of *P. aeruginosa* cultivability. Therefore, *P. aeruginosa* might be responsible for the protection of the whole dual consortia against ciprofloxacin activity. Based on the overall data, it can be speculated that reciprocal interferences occur between the different bacterial species in CF lung, regardless the level of oxygen. The findings also suggest that alterations of bacterial behavior due to species interplay may be important for disease progression in CF infection.

## Introduction

Cystic Fibrosis (CF) is a common lethal disease affecting nearly 70000 people around the world. It is characterized by the build-up of thick mucus overlying lung epithelial cells, wherein persistent cycles of chronic infection and inflammation occur (Gibson et al., 2003; Goss and Burns, 2007). The CF airways provide heterogeneous microenvironments containing variable levels of oxygen, pH, nutrients, and antibiotics. This heterogeneity contributes largely for the proliferation of a phylogenetically diverse ecosystem, influencing the consortia of microbes able to occupy it (Yang et al., 2011). Several key microbial species contribute to CF lung infection and disease progression, beginning early in life with *Staphylococcus aureus* and *Haemophilus influenzae* and culminating in chronic infections caused by *Pseudomonas aeruginosa* or *Burkholderia cepacia* complex species (Razvi et al., 2009; Price et al., 2013).

It is now recognized that the different bacteria coexisting in CF airways have mutual interactions and contribute to the pathogenesis of the disease (Hibbing et al., 2010; Rogers et al., 2010). Nonetheless, the precise ways under which the many different organisms interact within the CF airways, and how these interactions influence the behavior of the individual species, the activity of the polymicrobial communities, and the relationship between host and microbes are poorly understood. Some studies have highlighted the potentially important roles of such interspecies interactions in disease phenotype and clinical outcome of CF infections (Amin et al., 2010; Chattoraj et al., 2010; Bragonzi et al., 2012; Lopes et al., 2012; Twomey et al., 2012).

Because CF infection is no longer viewed as being caused by a single pathogen, antibiotics, often used to target a small group of species recognized as key CF pathogens, are generally ineffective when other atypical species are present (Lopes et al., 2012, 2014) failing in many cases (Leekha et al., 2011).

In this study, the reciprocal influence of *P. aeruginosa* with *S. aureus, S. maltophilia*, and *Inquilinus limosus* was assessed. The first three species are important opportunistic pathogens that are often multidrug resistant and contribute significantly to the disease progression (Döring and Hoiby, 2004; Hauser et al., 2011; Ciofu et al., 2013). *S. aureus* and *S. maltophilia* are commonly co-isolated with *P. aeruginosa* from CF respiratory cultures (Hoffman et al., 2006; Blau et al., 2014; Zemanick et al., 2015). It is, therefore, plausible to hypothesize that these species interact and that this could theoretically affect their virulence and persistence. *I. limosus* has been pointed as a potential threat for CF patients, mainly due to the mucoid physiology, the multidrug resistance pattern, and the ability to persist in the respiratory tract (Chiron et al., 2005). Therefore, it is aimed to evaluate the contribution of species interactions under variable-oxygen atmospheres, particularly among *P. aeruginosa* and the referred CF-associated species, in biofilm formation, phenotype, and in its response to antibiotherapy often used for CF lung infections treatment.

## Materials and Methods

### Bacterial Strains and Culture Conditions

*Pseudomonas aeruginosa* (wild-type strain UCBPP-PA14), *S. aureus* (wild-type strain ATCC 25923), *I. limosus* (strain M53, isolated from CF sputum), and *S. maltophilia* (isolated from CF sputum) were used throughout this work. *I. limosus* M53 was gently provided by Dr. Michael Surette (University of Calgary, Calgary, AB, Canada) (Sibley et al., 2011) and *S. maltophilia* by Dr. Joerg Steinmann (Institute of Medical Microbiology, University Hospital Essen, University of Duisburg-Essen, Germany) (Vidigal et al., 2013).

All strains were stored at -70 ± 2°C in tryptic soy broth (TSB, Liofilchem, Italy) supplemented with 20% of glycerol. Prior to each assay, bacteria were subcultured twice from frozen stock preparations onto TSB supplemented with 12% (w/v) of agar and incubated aerobically at 37°C for 24–48 h.

All assays were carried out by using a standardized bacterial inoculum. Briefly, an overnight culture was grown aerobically in TSB under agitation (120 rpm) at 37°C, being then adjusted with sterile broth medium to an OD_640_ corresponding to 1 × 10^7^ CFU/mL for all strains. For dual-species cultures, the suspended inoculums of each bacterial species were combined in a 1:1 ratio.

### Biofilm Formation under Aerobic and Anaerobic Environments

Each well of a 96-well polystyrene microtiter plate (Orange Scientific, Braine L’Alleud, Belgium) was seeded with 200 μL of standardized inoculum (single or dual cultures) and incubated at 37°C, 120 rpm, under aerobic and anaerobic environments. For aerobic assays, microtiter plates were placed in a standard incubator (n-biotek, Model NB-205Q, Korea). The anaerobic atmosphere was created in plastic boxes with AnaeroGen (Oxoid Limited, Hampshire, England). At different sampling time points, the liquid content of the microtiter plates was discarded and the wells were washed once with distilled sterile water. Biofilm formation was then assessed by plate counts and crystal violet (CV) assay as described below.

This experiment was performed in three independent assays for each one of the species used and conditions, and for a total of three combinations between species (*P. aeruginosa*+S. aureus; *P. aeruginosa*+*I. limosus*; and *P. aeruginosa*+*S. maltophilia*).

### Kinetics of Biofilm Formation

The kinetics of biofilm growth were performed every 2 h, until 24 h, through biofilm-cells cultivability.

Briefly, biofilm cells were detached by sonication using an ultrasound bath (Sonic model SC-52, UK), operating at 50 kHz, for 10 min, and then resuspended by pipetting up and down three times. This sonication step was previously optimized to ensure that all biofilm cells were detached from the wells of the microtiter plate, without cell disruption, and biofilms aggregates dispersed into single bacteria (Supplementary Figures 1 and 2).

Subsequently, the disrupted biofilms were serially diluted (1:10) in sterile water, streaked onto tryptic soy agar (TSA) plates and incubated at 37°C for 24 h, for total CFU counting. For dual-species biofilms, different selective agar media were used for better discrimination between the two species. *Pseudomonas* isolation agar (PIA) was used to assess *P. aeruginosa* counts. Mannitol Salt Agar (MSA) and *Burkholderia cepacia* selective agar (BCSA) supplemented with 300 000 IU/L polymyxin B and 100 mg/L ticarcillin are selective media commonly used to discriminate *S. aureus* and *I. limosus*, respectively. In dual biofilms, *S. maltophilia* viable cell counting was estimated by the difference between the average of total counts on TSA and the average of *P. aeruginosa* (planted on PIA). Afterward, selective agar plates were incubated at 37°C for 24 h. The specificity of the agar media was previous tested by growing each bacteria on the selective media of the other species and no growth was observed.

The number of cultivable bacterial cells in biofilms was determined and expressed per area of well (log CFU cm^-2^).

### Phenotype of 24 h-Old Biofilms

Single and dual species biofilms were allowed to grow for 24 h and, afterward, characterized in terms of their biomass and number of culturable cells, as follows:

#### Biomass

Biomass was quantified by CV staining method (Stepanovic et al., 2000). Briefly, wells were allowed air-drying for 10 min after washing. Attached bacteria were then fixed with methanol (Fisher Scientific, Leicestershire, UK) for 15 min and stained with 1% (vol/vol) CV (Merk, Germany) for 1 min. The excess stain was removed by aspirating the content of each well and washed twice with distilled sterile water. Lastly, wells were decolorized with 33% (vol/vol) of acetic acid (Fisher Scientific, UK) and the optical density (OD) of the obtained solution was measured at 570 nm using a microtiter plate reader (Model Sunrise-basic Tecan, Austria).

#### Cell Cultivability

The number of adhering bacteria was determined after biofilm cell detachment by sonication for 10 min, using an ultrasound bath, and then the viable cell count was carried out, as described above.

### Ciprofloxacin Activity against Single and Dual Biofilms

The antimicrobial action of ciprofloxacin was assessed against single and dual biofilms analysing cell cultivability. Briefly, each well of a 96-well polystyrene microtiter plate was seeded with 100 μL of bacterial culture (single or dual cultures), at 1 × 10^6^ CFU/mL, + 100 μL of ciprofloxacin at 1/4; × MIC, MIC or 4 × MIC determined for *P. aeruginosa*. The minimum inhibitory concentration (MIC) of ciprofloxacin (Sigma-Aldrich) for *P. aeruginosa* was 0.125 mg/L, as assessed by microdilution technique according to the EUCAST guidelines (EUCAST, 2003). Negative controls (CTRL) were also performed by adding 100 μL of TSB instead of the antibiotic. Microtiter plates were then incubated at 37°C, 120 rpm, under aerobic and anaerobic environments for 24 h.

The number of viable bacteria within biofilms was determined by CFU counting following the procedure abovementioned. Three independent assays were performed for each species and condition.

### Determination of the Competitive Index (CI) and the Relative Increase Ratio (RIR)

In dual cultures, the Competitive Index (CI) was defined as the *P. aeruginosa/S. aureus* or *P. aeruginosa/I. limosus* or *P. aeruginosa/ S. maltophilia* ratio within the output sample divided by the corresponding ratio in the inoculum (input): CI = (*P. aeruginosa*/*S. aureus* or *I. limosus* or *S. maltophilia*)_output_/(*P. aeruginosa*/*S. aureus* or *I. limosus* or *S. maltophilia*) _input_, where output and input samples were assessed after plating onto selective media serial dilutions of the sample taken at fixed times or the inoculum (*t* = 0), respectively, (Macho et al., 2007). For statistical purposes, CI values were first subjected to a Log transformation for normal distribution, then interpreted as follows: a CI value equal to 0 indicates equal competition of the two species; a positive CI value indicates a competitive advantage for *P. aeruginosa*; a negative CI value indicates a competitive advantage for *S. aureus* or *I. limosus* or *S. maltophilia*. Similarly to CI, the Relative Increase Ratio (RIR) was calculated based on the growth results obtained from monocultures of each strain (Macho et al., 2007).

### Statistical Analysis

Means and standard deviations are shown for each graph, derived from three independent assays. All statistical analyses were performed using Prism Software (GraphPad version 6.0 for Macintosh), considering as statistically significant a *p*-value less than 0.05.

Differences were assessed by ANOVA-test followed by Tukey multiple-comparison test for biofilm formation (aerobic *versus* anaerobic conditions and single *versus* mixed consortia single) and for ciprofloxacin activity (single *versus* mixed consortia). Regarding the kinetics of biofilm growth, statistical analysis was provided using Student’s *t*-test and the null hypothesis (single *versus* mixed consortia single). The latter was also used to inspect each CI and RIR indexes: the mean index was not significantly different from 1.0. When appropriate, CI and RIR from a given experiment were compared using unpaired Student’s *t*-test, and significant differences are suggestive of a meaningful competition between the species (Macho et al., 2007). Statistical significance is represented in figures by asterisks.

## Results

### Single and Dual-Species Biofilm Experiments under Variable Oxygen Conditions

It is well known that, in multispecies biofilms, the interactions established may encourage the coexistence (synergism) or, contrarily, confer advantage to one species, inhibiting the growth of other species (antagonism) (Harrison, 2007). In order to explain the possible interactions occurring between four CF-related pathogens in dual-species biofilms, several features were analyzed: the kinetic of biofilm formation in single and dual-species, the competitive indexes (CI and RIR), the phenotypic diversity (through cultivability assessment and biomass quantification) and the antimicrobial effect of ciprofloxacin on biofilm formation of single and dual-species.

**FIGURE 1:**
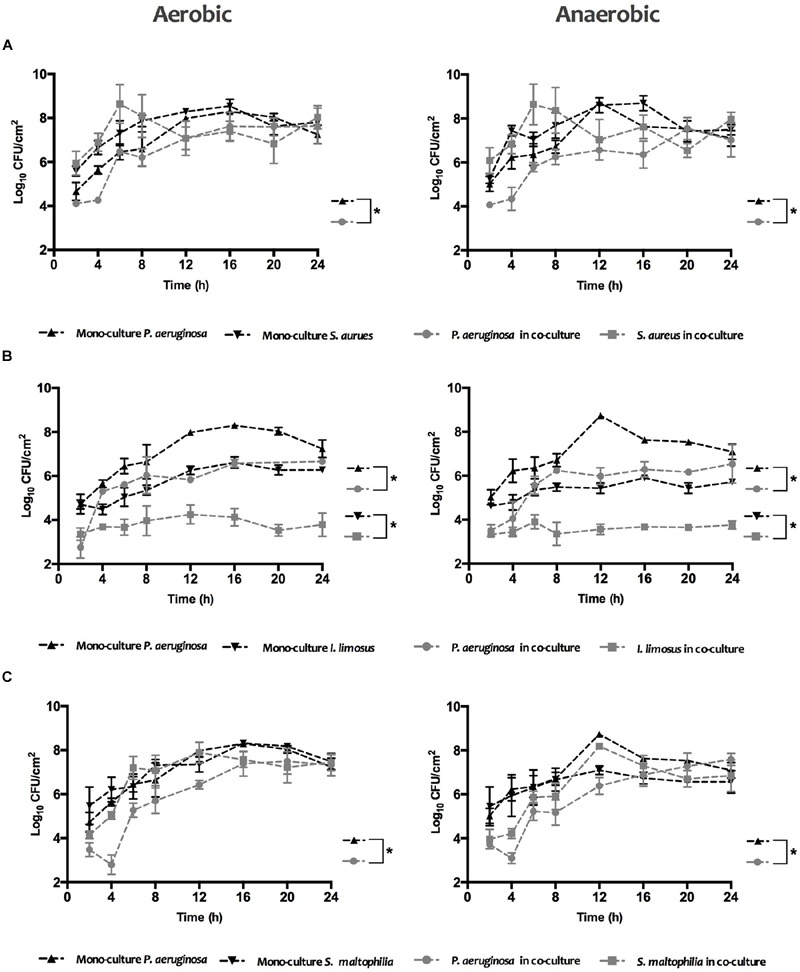
**Biofilm formation kinetics of (A)** *P. aeruginosa* and *S. aureus* **(B)** *P. aeruginosa* and *I. limosus*, and **(C)** *P. aeruginosa* and *S. maltophilia*, alone and in combination, under aerobic and anaerobic conditions. Data for each time point are shown as mean ± SD.

The biofilm growth under variable oxygen atmospheres for *S. aureus, I. limosus and S. maltophilia*, growing individually or in combination with *P. aeruginosa*, was assessed by CFU counts over 24 h (**Figure 1**). Concerning single cultures, similar growth trends were observed for aerobic and anaerobic environments with *P. aeruginosa* dominating for most cases (maximum of ∼Log 8.6 achieved). In dual cultures, the viability of each species in the consortium decreased in comparison with monocultures, being that *P. aeruginosa* prevailed over *I. limosus* in co-culture.

**FIGURE 2:**
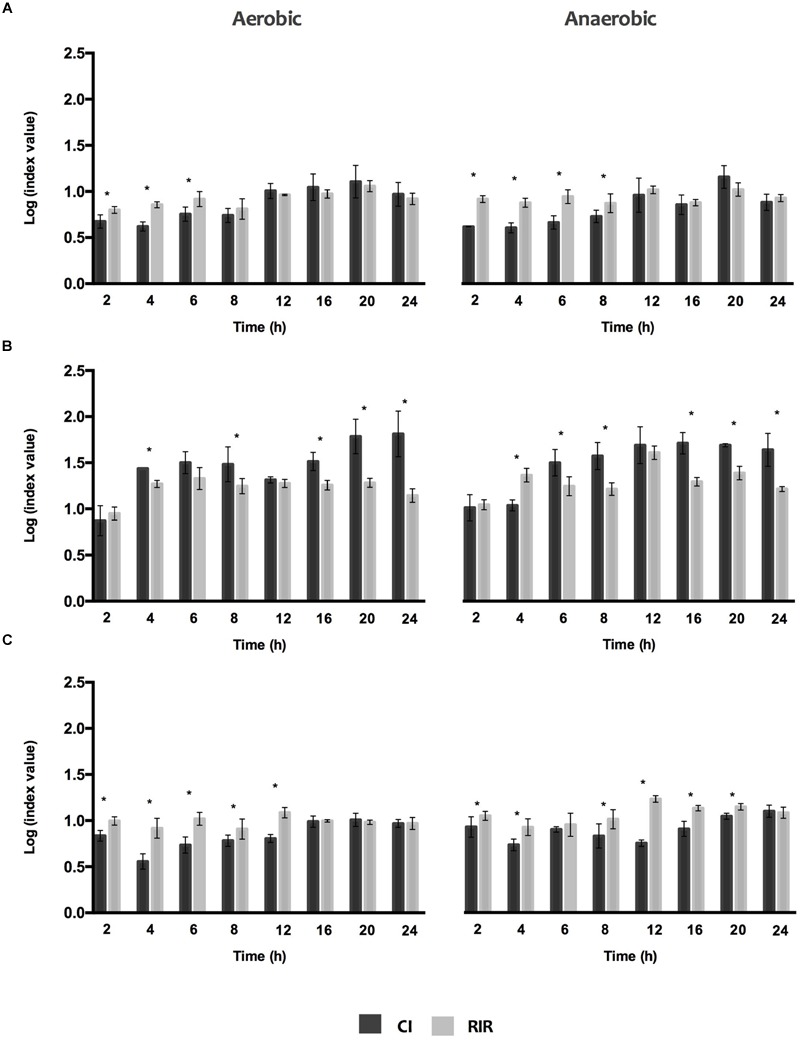
**Competitive index (CI) and Relative Increase Ratio (RIR) obtained for single and dual biofilm of (A)** *P. aeruginosa* and *S. aureus*, **(B)** *P. aeruginosa* and *I. limosus* and **(C)** *P. aeruginosa* and *S. maltophilia.* Data are shown as mean ± SD.

For a clear comprehension of the differences in the growth curves of each species in single *versus* dual cultures under the different O_2_ environments, CI and RIR indexes were estimated (**Figure 2**). Whilst CI allows comparing the differences among the growth curves of dual cultures, RIR index compares the growth curves of both species within pure cultures. As shown in **Figure 2**, a positive CI index is always observed, meaning a clear competitive advantage for *P. aeruginosa* over the other species in dual cultures. The dominant inhibitory effect of *P. aeruginosa* can be noticed in the lag and exponential phases (between 2 and 8 h), for *P. aeruginosa*+*S. aureus* dual cultures (**Figure 2A**) or even all over 24 h for *P. aeruginosa*+*I. limosus* and *P. aeruginosa+S. maltophilia* (**Figures 2B,C**). A strong inhibitory effect is observed for *P. aeruginosa*+*I. limosus* consortia, with statistical significances (*P* < 0.05) obtained for CI *versus* RIR for the 24 h of growth under both aerobic and anaerobic conditions.

**FIGURE 3:**
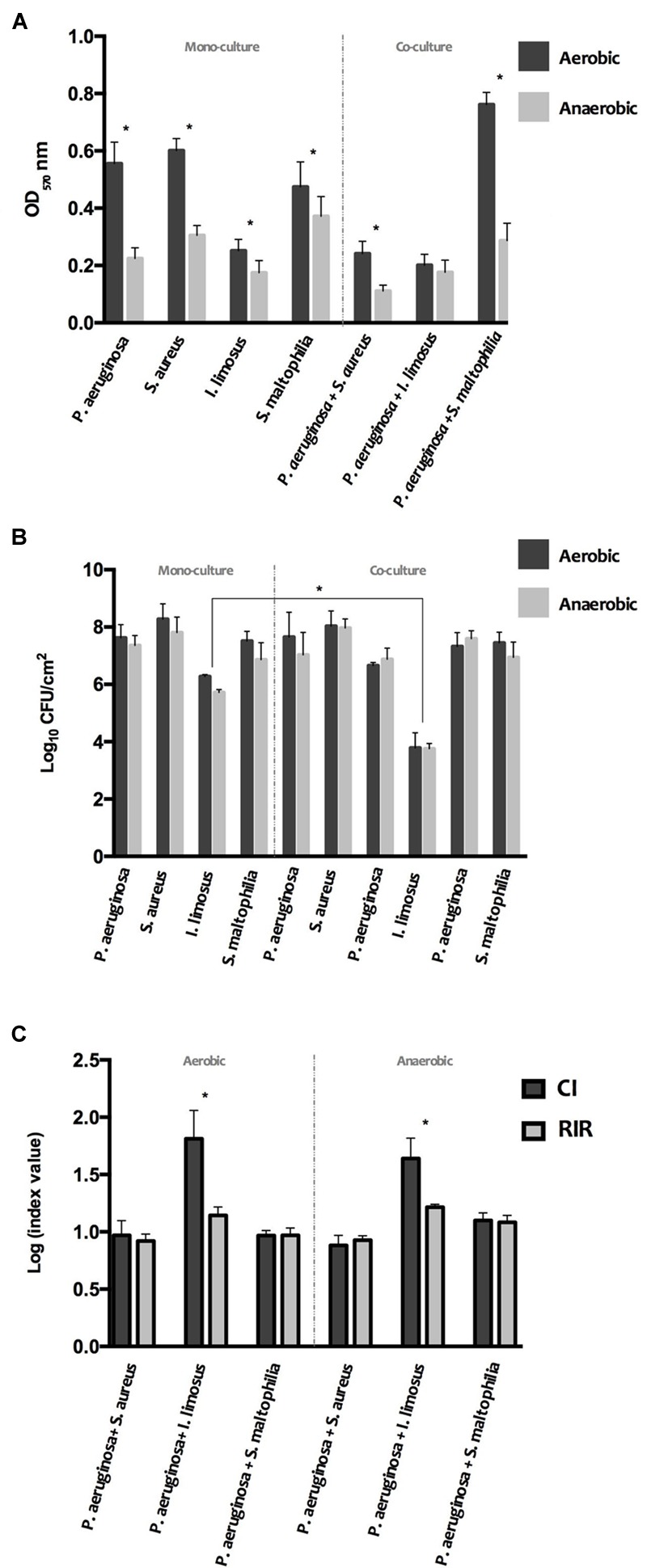
**Phenotype of single and dual 24 h-old biofilms, developed under aerobic and anaerobic conditions: (A)** biomass **(B)** and culturable cells. **(C)** Competitive Index (CI) and Relative Increase Ratio (RIR) obtained for dual biofilms in aerobic and anaerobic conditions. The results are shown as mean ± SD.

In order to assess whether species interactions could interfere with the biofilm-producing ability, the biomass and number of culturable cells of the 24 h-old dual biofilms formed under aerobic and anaerobic conditions were determined (**Figure 3**). Interactions among the species in the dual populations were evaluated by CI and RIR indexes. Data from single and dual cultures revealed that the amount of biofilm mass declined markedly under low oxygen environments (*P* < 0.05) for most cases (**Figure 3A**), however with no significant changes in terms of cell cultivability (**Figure 3B**). These results led to put the hypothesis that the species within biofilms are more prone to produce extracellular polymeric substances (EPS) when grown under aerobic atmospheres.

In the co-infection scenario, the number of viable cells of *P. aeruginosa* and *S. aureus* that adhered to polystyrene were not affected comparing with the respective single biofilms (**Figure 3B**), as confirmed by comparable CI and RIR values (**Figure 3C**). However, the biofilm mass level was significantly lower than that for both single populations (*P* < 0.05). Therefore, it can be speculated that the reciprocal interference observed in *P. aeruginosa* and *S. aureus* dual-biofilms may have led to a decrease on the EPS production by the overall consortium. Similarly to *P. aeruginosa*+*S. aureus* consortium, the cultivability of *P. aeruginosa*+*S. maltophilia* biofilms was not significantly disturbed, compared with single biofilms (**Figure 3B**), with CI *versus* RIR not showing significant differences among both bacterial species within the biofilm (**Figure 3C**). However, this reciprocal interference triggered an increase in the biomass of the overall consortium, in particular when developed aerobically (**Figure 3A**). Regarding *P. aeruginosa* and *I. limosus* consortia, dual populations produced lower biomass compared to *P. aeruginosa* single populations (*P* < 0.05), under aerobic environments (**Figure 3A**). These data can suggest a potential inhibition of EPS production by *P. aeruginosa* under this condition in the presence of *I. limosus*. Nonetheless, a significant decrease in *I. limosus* cultivability (**Figure 3B**) was observed (*P* < 0.05), under both conditions, with no significant disturbances for *P. aeruginosa* cultivability in dual populations (**Figure 3B**). The significant difference between CI and RIR indexes obtained for these dual cultures of *P. aeruginosa versus I. limosus* (*P* < 0.05) (**Figure 3C**), under both oxygen atmospheres, led to conclude that the loss of cultivability in *I. limosus* resulted from the interaction with *P. aeruginosa*, which outcompetes *I. limosus* and affects its growth in dual biofilms.

### Antimicrobial Activity of Ciprofloxacin on Single and Dual-Species Biofilm Formation

**FIGURE 4:**
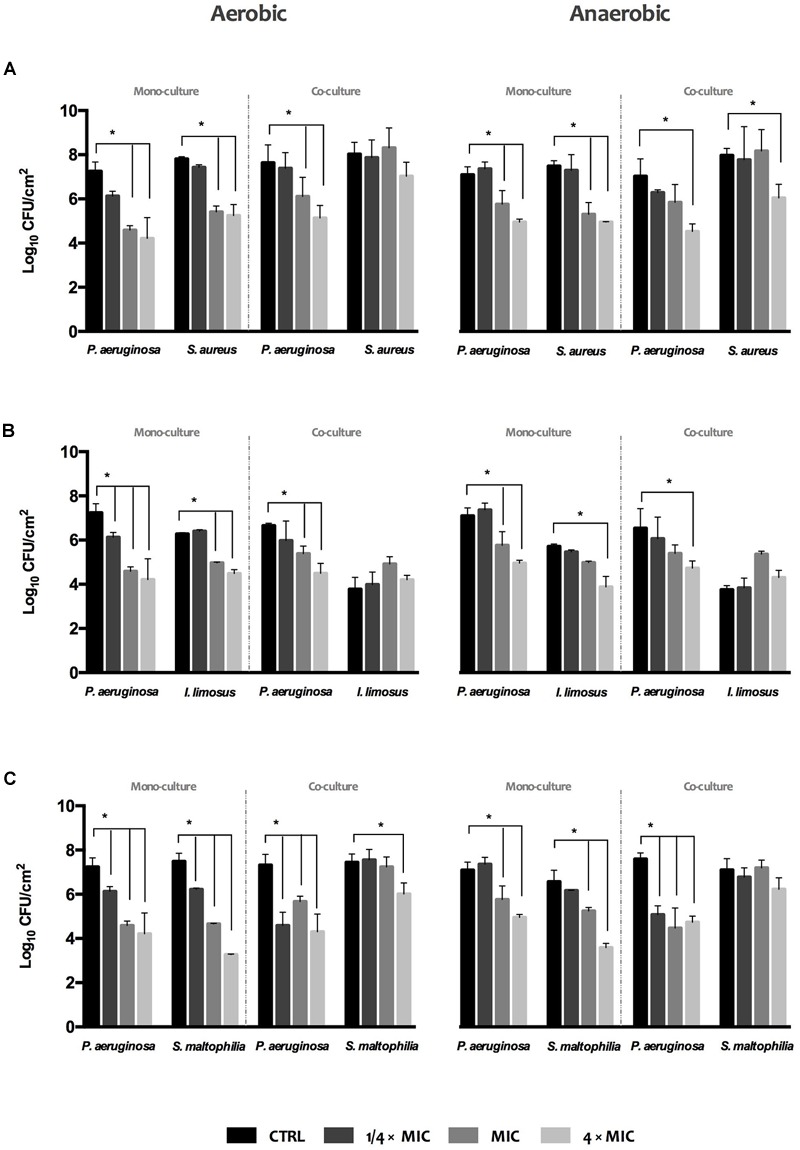
**Activity of ciprofloxacin against single and dual 24 h-old biofilms of (A)** *P. aeruginosa* and *S. aureus*, **(B)** *P. aeruginosa* and *I. limosus*, and **(C)** *P. aeruginosa* and *S. maltophilia*. The results are shown as mean ± SD.

The effect of ciprofloxacin at 1/4 × MIC, MIC and 4 × MIC (MIC = 0.125 mg/L, previously achieved for *P. aeruginosa*) on the development of biofilms under variable oxygen atmospheres was assessed by viable biofilm-cell count (**Figure 4**). Overall, increasing concentrations of antibiotic led to gradual reductions (maximum of ∼Log 3.3 achieved) of biofilm-cell viability in single and dual cultures, both under aerobic and anaerobic conditions, when compared to un-treated biofilms (CTRL). In turn, viability reduction was not so evident when ciprofloxacin was used against polymicrobial biofilms. When *P. aeruginosa* grown in dual biofilms with *S. aureus* or *S. maltophilia*, ciprofloxacin was only effective on *P. aeruginosa* (**Figures 4A,C**), with increasing antibiotic doses leading to drastic inhibition of *P. aeruginosa* cultivability. Regarding *P. aeruginosa*+*I. limosus* consortium, the viability of *I. limosus* remains the same or increases relatively to the un-treated consortium and its single biofilm, highlighting loss of susceptibility toward ciprofloxacin (**Figure 4B**).

Together, these findings revealed that *S. aureus, I. limosus*, and *S.* maltophilia, when grown in consortia with *P. aeruginosa*, seem to be overprotected by it against ciprofloxacin action. In fact, these species showed increasing tolerance to ciprofloxacin in comparison with the corresponding single species biofilms and with un-treated biofilms.

## Discussion

The complex microbial communities of the CF respiratory tract constitute a challenging niche in which a number of microbial species can interact, contributing to disease progression and clinical outcome. The significance of microbe-microbe interactions in CF infections and the consequences of such interplay remain poorly understood, thus studies helping to comprehend the role that species interactions may play in infection prevalence and disease progression are welcome. Thus, in the this study, it was aimed to gain insights into the role of bacterial interactions, in particular among *P. aeruginosa* with other CF-associated species (*S. aureus, I. limosus*, and *S. maltophilia)*, in biofilm formation and phenotype and in driving a response to antibiotherapy, often used for CF lung infections treatment, under variable-oxygen atmospheres.

Unlike *P. aeruginosa*, which has been extensively studied under similar CF environments (Field et al., 2005; Hogardt and Heesemann, 2010; King et al., 2010; Schertzer et al., 2010; Schobert and Jahn, 2010), the behavior of other bacterial species under the oxygen conditions found in *in vivo* CF airways are still poorly understood, often failing in consider the role of biofilm development, oxygen availability, and the interplay among microorganisms within polymicrobial infections in CF context.

The biofilm formation kinetics, under aerobic and anaerobic conditions, and the significant differences between the CI and RIR indexes indicated a competitive advantage of *P. aeruginosa* over the remaining species, when in dual biofilms. This competition was observed during the first stages of biofilm growth (lag and exponential growth phases) or even for all over 24 h, resulting in higher numbers of *P. aeruginosa* compared to *S. aureus, I. limosus*, and *S. maltophilia* cells. In fact, antagonism between microorganisms within a community can be a result of bacterial competition for both nutrients and space, or to direct antagonistic effects (Harrison, 2007; Hibbing et al., 2010). There is evidence supporting antagonism between *P. aeruginosa* and *S. aureus* as it has been reported that *S. aureus* is susceptible to *P. aeruginosa* exoproducts, such as pyocyanin, hydrogen cyanide or alkyl-hydroxyquinoline *N*-oxides (HQNO), which are able to suppress its aerobic metabolism and growth (Hoffman et al., 2006; Biswas et al., 2009). Recently, Filkins et al. (2015) reinforced that HQNO and siderophore produced by *P. aeruginosa* additively induce a transition of *S. aureus* metabolism from aerobic respiration to fermentation and eventually lead to loss of *S. aureus* viability.

Interestingly, when simultaneous cultured in dual biofilms, *I. limosus* and *S. maltophilia* cannot coexist with *P. aeruginosa* in a dynamic equilibrium. The results from CI *versus* RIR suggested that *I. limosus* and *S. maltophilia* are, in a general point of view, outcompeted. These findings are in agreement with Pompilio et al. (2015) that found that *P. aeruginosa*, in dual biofilms, significantly affect *S. maltophilia* growth. They also shown that, when grown with *S. maltophilia* in dual biofilms, some *P. aeruginosa* virulence factors, as alkaline protease and alginate, were up-regulated.

An important feature of *P. aeruginosa* infection is its ability to form biofilms, which is one of the contributing factors to reduce antibiotic efficacy and provide tolerance to the host inflammatory defense mechanism (Høiby et al., 2011). Data obtained from single and dual cultures showed that the biofilm-producing ability was markedly higher under aerobic atmospheres. Kadouri and Tran (2013) measured biofilm formation by a variety of opportunistic pathogens, at different oxygen concentrations, and also established a positive correlation between oxygen levels and biofilm formation ability. Data also suggested that the changes in the overall biofilm mass and culturable cells of dual biofilms can be due to reciprocal interference between species in the consortia or even only triggered by *P. aeruginosa*. Dual biofilms encompassing *P. aeruginosa*+*S. aureus* or *P. aeruginosa*+*S. maltophilia* were not significantly affected in terms of biofilm-cell number, as the whole consortia had similar proportions of both species (∼50% each), for aerobic and anaerobic atmospheres. Nonetheless, the reciprocal species interference (CI *versus* RIR without significant differences) led to marked changes in biofilm mass of the final consortia, suggesting that EPS production changes may occur. In chronic CF, the matrix of mucoid *P. aeruginosa* biofilms is majority constituted by alginate, a linear polyanionic EPS (Bjarnsholt et al., 2009), that has shown to significantly contribute to decreased susceptibility of biofilms to antibiotic treatment and to human antibacterial defense mechanisms (Pier et al., 2001). For instance, the production of biofilm biomass by *P. aeruginosa*+*S. aureus* was significantly reduced under both oxygen environments whereas an increase in EPS accumulation was observed for *P. aeruginosa*+*S. maltophilia* biofilms, in particular under aerobic conditions. In apparent contradiction with the overview of the results obtained from kinetic data, that revealed a clear dominant advantage for *P. aeruginosa*, biofilm-formation ability of dual cultures seems to result from a reciprocal interference between both species in *P. aeruginosa*+*S. aureus* and *P. aeruginosa*+*S. maltophilia* consortia. These findings could be explained considering the complexity of the microbial interactions in the CF lung, and the presence of many factors contributing to the biofilm formation.

Concerning *P. aeruginosa*+*I. limosus* biofilms, results demonstrated that this biofilm was significantly disturbed in terms of overall biomass that could be justified by the predominance of *P. aeruginosa* in the consortium. A significant difference among CI *versus* RIR indexes under both oxygen conditions led to conclude that a strong competition between the species occurred, with *P. aeruginosa* predominating and outcompeting *I. limosus*. In a previous work, Lopes et al. (2012) has demonstrated that, in dual biofilms, *P. aeruginosa* biomass was markedly reduced by the presence of *I. limosus*.

Understanding the interspecies interactions in dual infections is crucial, not only because they can modulate the virulence and persistence of pathogens, but also because that knowledge can assist the design of tailored therapy regimens and the definition of new antimicrobial agents, new targets, and strategies for CF disease control. Ciprofloxacin is one of the most commonly used oral agent used to control pulmonary infections caused by *P. aeruginosa* in CF patients (Bittar and Rolain, 2010; Guss et al., 2011; Sriramulu, 2013). The exposure of biofilms to increasing concentrations of ciprofloxacin lead to gradual inhibition in biofilm cells, for both aerobic and anaerobic environments. Furiga et al. (2015), when assessing the efficacy of a new quorum sensing inhibitor in combination with ciprofloxacin, against *P. aeruginosa* biofilms, observed a synergistic anti-biofilm activity regardless the oxygen conditions. When applied to dual consortia, ciprofloxacin only reduced *P. aeruginosa* viability, being the viability of the other species of the consortia unchanged. Data suggest a potential protective effect of *P. aeruginosa* over the other species against ciprofloxacin. Similar hints were pointed out by Pompilio et al. (2015) as they stated that *P. aeruginosa* might be responsible for the protection of *S. maltophilia* against tobramycin in dual biofilms due to alginate overproducing. Moreover, data also shown that, independently of the reduced biomass noticed in *P. aeruginosa*+*S. aureus* and *P. aeruginosa*+*I. limosus* dual biofilms, the high number of biofilm-encased cells was enough to imply an increased tolerance on those consortia. In fact, biofilm tolerance is thought to be multifactorial, resulting by (i) decreased growth rates, due to oxygen and nutrient microscale heterogeneities within the biofilm; (ii) the protective barrier provided by the EPS, retarding or inactivating the penetration of antibiotics into the biofilm; (iii) the number and spatial distribution of bacterial cells within biofilms; (iv) the expression of biofilm-specific resistance genes; (vi) the presence of “persisters”, i.e., a subpopulation of microorganisms that differentiate into a dormant and protected state, like a spore-bacterial form (Hill et al., 2005; Hassett et al., 2010).

The majority of studies about interactions in the polymicrobial CF community focus on the traditional pathogen *P. aeruginosa*, due to its prevalence in CF lung, examining its ability to form biofilms, as this lifestyle protects the microorganism to the host responses and to numerous antibiotics, and its potential do develop chronic infections (Magalhães et al., 2015). However, several studies have highlighted the crucial role of interspecies interactions in influencing infection status, clinical outcomes, and response to therapy in CF infections, suggesting that the role of other microbial species needs to be considered (Harrison, 2007; Ryan et al., 2008; Sibley et al., 2008; Rogers et al., 2010). In the present work, *P. aeruginosa* exhibited a dominant inhibitory effect, when co-cultured in biofilms with *S. aureus, I. limosus*, and *S. maltophilia* under both aerobic and anaerobic conditions. On the other hand, biofilm formation ability results from the reciprocal interaction between *P. aeruginosa* and *S. aureus* or *S. maltophilia*. Furthermore, it was found that the exposure of dual biofilms to ciprofloxacin reduced the viability of *P. aeruginosa* but not of *S. aureus, I. limosus*, and *S. maltophilia.* These findings suggest that *P. aeruginosa* might be responsible for the protection of *S. aureus, I. limosus*, and *S. maltophilia*, in dual biofilms toward ciprofloxacin In conclusion, this study underlines the importance of bacterial interactions in lung infections and in particular the complexity of the interactions of different pathogens that coexist in the CF airways.

Nonetheless, further investigations are required for inspecting the role of the EPS matrix in the behavior the polymicrobial consortia studied and considering host response and the molecular mechanisms involved in the microbe-microbe interactions, using both *in vitro* and *in vivo* models of chronic infection that better mirror the progression of CF lung infections.

## Author Contributions

Conceived and designed the experiments: SPL and MOP. Performed the experiments: APM and SPL. Analysed the data: APM, SPL, and MOP. Wrote the paper: APM, SPL, and MOP.

## Conflict of Interest Statement

The authors declare that the research was conducted in the absence of any commercial or financial relationships that could be construed as a potential conflict of interest.

## References

[B1] Amin R. Dupuis A. Aaron S. D. Ratjen F. (2010). The effect of chronic infection with *Aspergillus fumigatus* on lung function and hospitalization in patients with cystic fibrosis. *Chest* 137 171–176. 10.1378/chest.09-1103 19567494

[B2] Biswas L. Biswas R. Schlag M. Bertram R. Götz F. (2009). Small-colony variant selection as a survival strategy for *Staphylococcus aureus* in the presence of *Pseudomonas aeruginosa*. *Appl. Environ. Microbiol.* 75 6910–6912. 10.1128/AEM.01211-09 19717621 PMC2772425

[B3] Bittar F. Rolain J.-M. (2010). Detection and accurate identification of new or emerging bacteria in cystic fibrosis patients. *Clin. Microbiol. Infect.* 16 809–820. 10.1111/j.1469-0691.2010.03236.x 20880410

[B4] Bjarnsholt T. Jensen P. Ø Fiandaca M. J. Pedersen J. Hansen C. R. Andersen C. B. (2009). *Pseudomonas aeruginosa* biofilms in the respiratory tract of cystic fibrosis patients. *Pediatr. Pulmonol.* 44 547–558. 10.1002/ppul.21011 19418571

[B5] Blau H. Linnane B. Carzino R. Tannenbaum E.-L. Skoric B. Robinson P. J. (2014). Induced sputum compared to bronchoalveolar lavage in young, non-expectorating cystic fibrosis children. *J. Cyst. Fibros.* 13 106–110. 10.1016/j.jcf.2013.05.013 23806622

[B6] Bragonzi A. Farulla I. Paroni M. Twomey K. B. Pirone L. Lorè N. I. (2012). Modelling Co-infection of the cystic fibrosis lung by *Pseudomonas aeruginosa* and *Burkholderia cenocepacia* reveals influences on biofilm formation and host response. *PLoS ONE* 7:e52330 10.1371/journal.pone.0052330 PMC3528780 23284990

[B7] Chattoraj S. S. Murthy R. Ganesan S. Goldberg J. B. Zhao Y. Hershenson M. B. (2010). *Pseudomonas aeruginosa* alginate promotes *Burkholderia cenocepacia* persistence in cystic fibrosis transmembrane conductance regulator knockout mice. *Infect. Immun.* 78 984–993. 10.1128/IAI.01192-09 20048042 PMC2825924

[B8] Chiron R. Marchandin H. Counil F. Jumas-Bilak E. Freydière A.-M. Bellon G. (2005). Clinical and microbiological features of *Inquilinus* sp. isolates from five patients with cystic fibrosis. *J. Clin. Microbiol.* 43 3938–3943. 10.1128/JCM.43.8.3938-3943.2005 16081934 PMC1233925

[B9] Ciofu O. Hansen C. R. Høiby N. (2013). Respiratory bacterial infections in cystic fibrosis. *Curr. Opin. Pulm. Med.* 19 251–258. 10.1097/MCP.0b013e32835f1afc 23449384

[B10] Döring G. Hoiby N. (2004). Early intervention and prevention of lung disease in cystic fibrosis: a European consensus. *J. Cyst. Fibros.* 3 67–91. 10.1016/j.jcf.2004.03.008 15463891

[B11] EUCAST (2003). Determination of minimum inhibitory concentrations (MICs) of antibacterial agents by broth dilution. *Clin. Microbiol. Infect.* 9 ix–xv. 10.1046/j.1469-0691.2003.00790.x 11168187

[B12] Field T. R. White A. Elborn J. S. Tunney M. M. (2005). Effect of oxygen limitation on the in vitro antimicrobial susceptibility of clinical isolates of *Pseudomonas aeruginosa* grown planktonically and as biofilms. *Eur. J. Clin. Microbiol. Infect. Dis.* 24 677–687. 10.1007/s10096-005-0031-9 16249934

[B13] Filkins L. M. Graber J. A. Olson D. G. Dolben E. L. Lynd L. R. Bhuju S. (2015). Coculture of *Staphylococcus aureus* with *Pseudomonas aeruginosa* drives *S. aureus* towards fermentative metabolism and reduced viability in a cystic fibrosis model. *J. Bacteriol.* 197 2252–2264. 10.1128/JB.00059-15 25917910 PMC4524177

[B14] Furiga A. Lajoie B. El Hage S. Baziard G. Roques C. (2015). Impairment of *Pseudomonas aeruginosa* biofilm resistance to antibiotics by combination with a new quorum sensing inhibitor. *Antimicrob. Agents Chemother.* 60 1676–1686. 10.1128/AAC.02533-15 26711774 PMC4775964

[B15] Gibson R. L. Burns J. L. Ramsey B. W. (2003). Pathophysiology and management of pulmonary infections in cystic fibrosis. *Am. J. Respir. Crit. Care Med.* 168 918–951. 10.1164/rccm.200304-505SO 14555458

[B16] Goss C. H. Burns J. L. (2007). Exacerbations in cystic fibrosis. 1: epidemiology and pathogenesis. *Thorax* 62 360–367. 10.1136/thx.2006.060889 17387214 PMC2092469

[B17] Guss A. M. Roeselers G. Newton I. L. G. Young C. R. Klepac-Ceraj V. Lory S. (2011). Phylogenetic and metabolic diversity of bacteria associated with cystic fibrosis. *ISME J.* 5 20–29. 10.1038/ismej.2010.88 20631810 PMC3105664

[B18] Harrison F. (2007). Microbial ecology of the cystic fibrosis lung. *Microbiology* 153 917–923. 10.1099/mic.0.2006/004077-0 17379702

[B19] Hassett D. J. Korfhagen T. R. Irvin R. T. Schurr M. J. Sauer K. Lau G. W. (2010). *Pseudomonas aeruginosa* biofilm infections in cystic fibrosis: insights into pathogenic processes and treatment strategies. *Expert Opin. Ther. Targets* 14 117–130. 10.1517/14728220903454988 20055712

[B20] Hauser A. R. Jain M. Bar-Meir M. McColley S. A. (2011). Clinical significance of microbial infection and adaptation in cystic fibrosis. *Clin. Microbiol. Rev.* 24 29–70. 10.1128/CMR.00036-10 21233507 PMC3021203

[B21] Hibbing M. E. Fuqua C. Parsek M. R. Peterson S. B. (2010). Bacterial competition: surviving and thriving in the microbial jungle. *Nat. Rev. Microbiol.* 8 15–25. 10.1038/nrmicro2259 19946288 PMC2879262

[B22] Hill D. Rose B. Pajkos A. Robinson M. Bye P. Bell S. (2005). Antibiotic susceptabilities of *Pseudomonas aeruginosa* isolates derived from patients with cystic fibrosis under aerobic, anaerobic, and biofilm conditions. *J. Clin. Microbiol.* 43 5085–5090. 10.1128/JCM.43.10.5085-5090.2005 16207967 PMC1248524

[B23] Hoffman L. R. Déziel E. D’Argenio D. A. Lépine F. Emerson J. McNamara S. (2006). Selection for *Staphylococcus aureus* small-colony variants due to growth in the presence of *Pseudomonas aeruginosa*. *Proc. Natl. Acad. Sci. U.S.A.* 103 19890–19895. 10.1073/pnas.0606756104 17172450 PMC1750898

[B24] Hogardt M. Heesemann J. (2010). Adaptation of *Pseudomonas aeruginosa* during persistence in the cystic fibrosis lung. *Int. J. Med. Microbiol.* 300 557–562. 10.1016/j.ijmm.2010.08.008 20943439

[B25] Høiby N. Ciofu O. Johansen H. K. Song Z. Moser C. Jensen P. Ø, (2011). The clinical impact of bacterial biofilms. *Int. J. Oral Sci.* 3 55–65. 10.4248/IJOS11026 21485309 PMC3469878

[B26] Kadouri D. E. Tran A. (2013). Measurement of predation and biofilm formation under different ambient oxygen conditions using a simple gasbag-based system. *Appl. Environ. Microbiol.* 79 5264–5271. 10.1128/AEM.01193-13 23811501 PMC3753949

[B27] King P. Citron D. M. Griffith D. C. Lomovskaya O. Dudley M. N. (2010). Effect of oxygen limitation on the in vitro activity of levofloxacin and other antibiotics administered by the aerosol route against *Pseudomonas aeruginosa* from cystic fibrosis patients. *Diagn. Microbiol. Infect. Dis.* 66 181–186. 10.1016/j.diagmicrobio.2009.09.009 19828274

[B28] Leekha S. Terrell C. L. Edson R. S. (2011). General principles of antimicrobial therapy. *Mayo Clin. Proc.* 86 156–167. 10.4065/mcp.2010.0639 21282489 PMC3031442

[B29] Lopes S. P. Azevedo N. F. Pereira M. O. (2014). Emergent bacteria in cystic fibrosis: in vitro biofilm formation and resilience under variable oxygen conditions. *Biomed. Res. Int.* 2014:678301 10.1155/2014/678301 PMC4020565 24868541

[B30] Lopes S. P. Ceri H. Azevedo N. F. Pereira M. O. (2012). Antibiotic resistance of mixed biofilms in cystic fibrosis: impact of emerging microorganisms on treatment of infection. *Int. J. Antimicrob. Agents* 40 260–263. 10.1016/j.ijantimicag.2012.04.020 22770521

[B31] Macho A. P. Zumaquero A. Ortiz-Martín I. Beuzón C. R. (2007). Competitive index in mixed infections: a sensitive and accurate assay for the genetic analysis of *Pseudomonas syringae*-plant interactions. *Mol. Plant Pathol.* 8 437–450. 10.1111/j.1364-3703.2007.00404.x 20507512

[B32] Magalhães A. P. Azevedo N. F. Pereira M. O. Lopes S. P. (2015). The cystic fibrosis microbiome in an ecological perspective and its impact in antibiotic therapy. *Appl. Microbiol. Biotechnol.* 100 1163–1181. 10.1007/s00253-015-7177-x 26637419

[B33] Pier G. B. Coleman F. Grout M. Franklin M. Ohman D. E. (2001). Role of alginate O acetylation in resistance of mucoid *Pseudomonas aeruginosa* to opsonic phagocytosis. *Infect. Immun.* 69 1895–1901. 10.1128/IAI.69.3.1895-1901.2001 11179370 PMC98099

[B34] Pompilio A. Crocetta V. De Nicola S. Verginelli F. Fiscarelli E. Di Bonaventura G. (2015). Cooperative pathogenicity in cystic fibrosis: *Stenotrophomonas maltophilia* modulates *Pseudomonas aeruginosa* virulence in mixed biofilm. *Front. Microbiol.* 6:951 10.3389/fmicb.2015.00951 PMC4584994 26441885

[B35] Price K. E. Hampton T. H. Gifford A. H. Dolben E. L. Hogan D. A. Morrison H. G. (2013). Unique microbial communities persist in individual cystic fibrosis patients throughout a clinical exacerbation. *Microbiome* 1:27 10.1186/2049-2618-1-27 PMC3971630 24451123

[B36] Razvi S. Quittell L. Sewall A. Quinton H. Marshall B. Saiman L. (2009). Respiratory microbiology of patients with cystic fibrosis in the United States, 1995 to 2005. *Chest* 136 1554–1560. 10.1378/chest.09-0132 19505987

[B37] Rogers G. B. Hoffman L. R. Whiteley M. Daniels T. W. V. Carroll M. P. Bruce K. D. (2010). Revealing the dynamics of polymicrobial infections: implications for antibiotic therapy. *Trends Microbiol.* 18 357–364. 10.1016/j.tim.2010.04.005 20554204 PMC3034215

[B38] Ryan R. P. Fouhy Y. Garcia B. F. Watt S. A. Niehaus K. Yang L. (2008). Interspecies signalling via the *Stenotrophomonas maltophilia* diffusible signal factor influences biofilm formation and polymyxin tolerance in *Pseudomonas aeruginosa*. *Mol. Microbiol.* 68 75–86. 10.1111/j.1365-2958.2008.06132.x 18312265

[B39] Schertzer J. W. Brown S. A. Whiteley M. (2010). Oxygen levels rapidly modulate *Pseudomonas aeruginosa* social behaviours via substrate limitation of PqsH. *Mol. Microbiol.* 77 1527–1538. 10.1111/j.1365-2958.2010.07303.x 20662781 PMC3098721

[B40] Schobert M. Jahn D. (2010). Anaerobic physiology of *Pseudomonas aeruginosa* in the cystic fibrosis lung. *Int. J. Med. Microbiol.* 300 549–556. 10.1016/j.ijmm.2010.08.007 20951638

[B41] Sibley C. D. Duan K. Fischer C. Parkins M. D. Storey D. G. Rabin H. R. (2008). Discerning the complexity of community interactions using a *Drosophila* model of polymicrobial infections. *PLoS Pathog.* 4:e1000184 10.1371/journal.ppat.1000184 PMC2566602 18949036

[B42] Sibley C. D. Grinwis M. E. Field T. R. Eshaghurshan C. S. Faria M. M. Dowd S. E. (2011). Culture enriched molecular profiling of the cystic fibrosis airway microbiome. *PLoS ONE* 6:e22702 10.1371/journal.pone.0022702 PMC3145661 21829484

[B43] Sriramulu D. (2013). Evolution and impact of bacterial drug resistance in the context of cystic fibrosis disease and nosocomial settings. *Microbiol. Insights* 6 29–36. 10.4137/MBI.S10792 24826072 PMC3987750

[B44] Stepanovic S. Vukovic D. Dakic I. Savic B. Svabic-Vlahovic M. (2000). A modified microtiter-plate test for quantification of staphylococcal biofilm formation. *J. Microbiol. Methods* 40 175–179. 10.1016/S0167-7012(00)00122-6 10699673

[B45] Twomey K. B. O’Connell O. J. McCarthy Y. Dow J. M. O’Toole G. A. Plant B. J. (2012). Bacterial cis-2-unsaturated fatty acids found in the cystic fibrosis airway modulate virulence and persistence of *Pseudomonas aeruginosa*. *ISME J.* 6 939–950. 10.1038/ismej.2011.167 22134647 PMC3329111

[B46] Vidigal P. G. Schmidt D. Stehling F. Mellies U. Steinmann E. Buer J. (2013). Development of a quantitative immunofluorescence assay for detection of *Stenotrophomonas maltophilia* antibodies in patients with cystic fibrosis. *J. Cyst. Fibros.* 12 651–654. 10.1016/j.jcf.2013.04.011 23706828

[B47] Yang L. Jelsbak L. Molin S. (2011). Microbial ecology and adaptation in cystic fibrosis airways. *Environ. Microbiol.* 13 1682–1689. 10.1111/j.1462-2920.2011.02459.x 21429065

[B48] Zemanick E. T. Wagner B. D. Robertson C. E. Stevens M. J. Szefler S. J. Accurso F. J. (2015). Assessment of airway microbiota and inflammation in cystic fibrosis using multiple sampling methods. *Ann. Am. Thorac. Soc.* 12 221–229. 10.1513/AnnalsATS.201407-310OC 25474078 PMC4342834

