# Supplementary material for: Insights into Cystic Fibrosis Polymicrobial Consortia: The Role of Species Interactions in Biofilm Development, Phenotype, and Response to In-Use Antibiotics
Source: Front Microbiol. 2017 Jan 13;7:2146. doi: 10.3389/fmicb.2016.02146 (PMC5233685; doi:10.3389/fmicb.2016.02146)
Supplement: Supplementary file 1 [file Image_1.pdf]

Supplementary Material

Insights into cystic fibrosis polymicrobial consortia: the role of species interactions in biofilm development, phenotype and response to in-use antibiotics.

Andreia P. Magalhães*, Susana P. Lopes and Maria O. Pereira

*** Correspondence:** amagalhaes@ceb.uminho.pt

1 Supplementary Figures

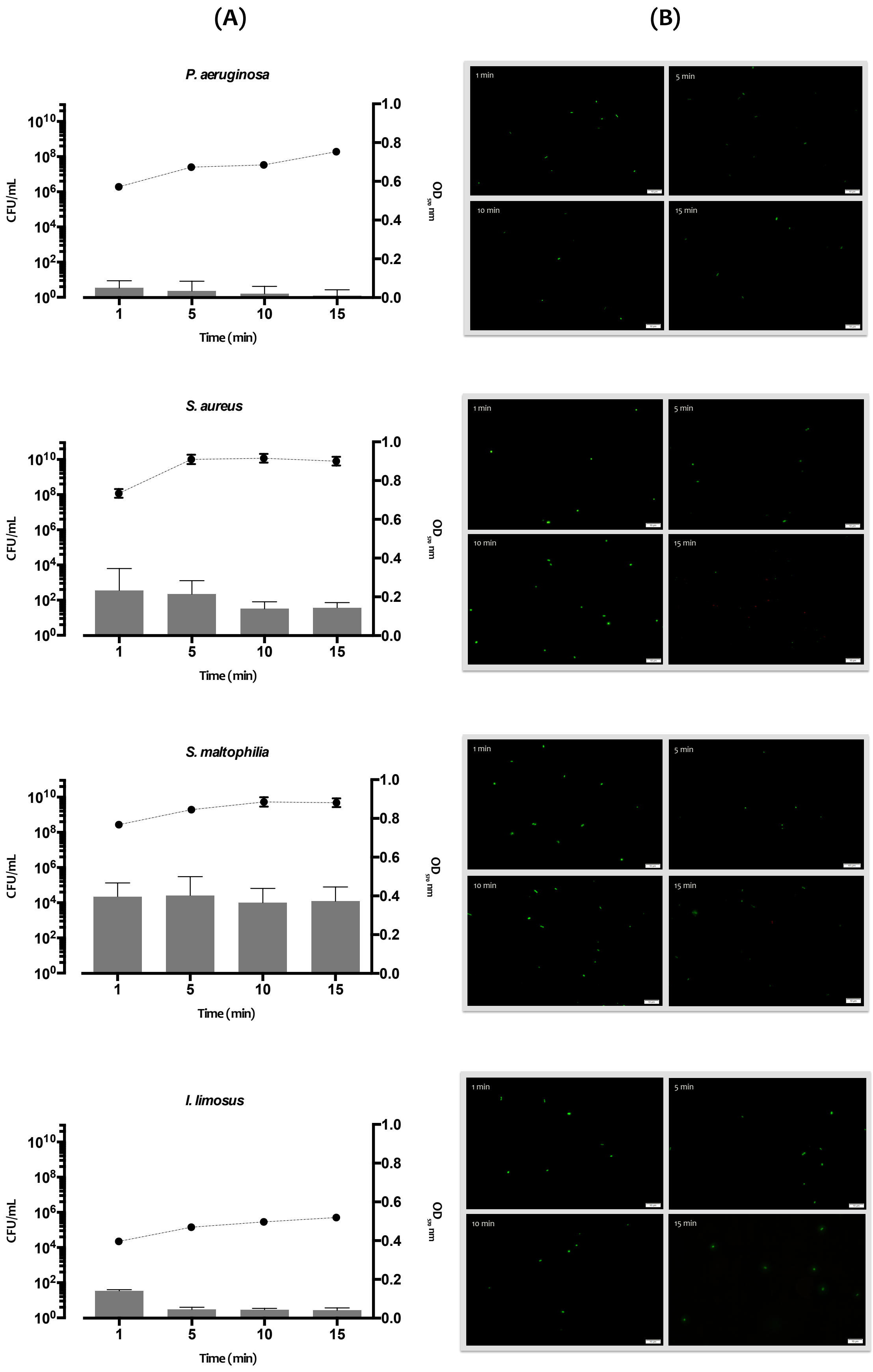
(A)
(B)
P. aeruginosa
S. aureus
S. maltophilia
I. limosus
CFU/mL
OD
Time (min)
1 min
5 min
10 min
15 min

Supplementary Figure 1. Time optimization for 24 h-old biofilm cell detachment in mono-cultures of *P. aeruginosa, S. aureus, S. maltophilia* and *I. limosus* under aerobic conditions. **(A)** Biomass (gray bars) and culturable cells (black line) and **(B)** cell viability using SYTO BC/PI (ThermoFisher Scientific) as a LIVE/ DEAD cell viability kit.
