# Supplementary material for: Insights into Cystic Fibrosis Polymicrobial Consortia: The Role of Species Interactions in Biofilm Development, Phenotype, and Response to In-Use Antibiotics
Source: Front Microbiol. 2017 Jan 13;7:2146. doi: 10.3389/fmicb.2016.02146 (PMC5233685; doi:10.3389/fmicb.2016.02146)
Supplement: Supplementary file 2 [file Image_2.pdf]

Supplementary Material

Insights into cystic fibrosis polymicrobial consortia: the role of species interactions in biofilm development, phenotype and response to in-use antibiotics.

Andreia P. Magalhães*, Maria O. Pereira and Susana P. Lopes

*** Correspondence:** amagalhaes@ceb.uminho.pt

1 Supplementary Figures

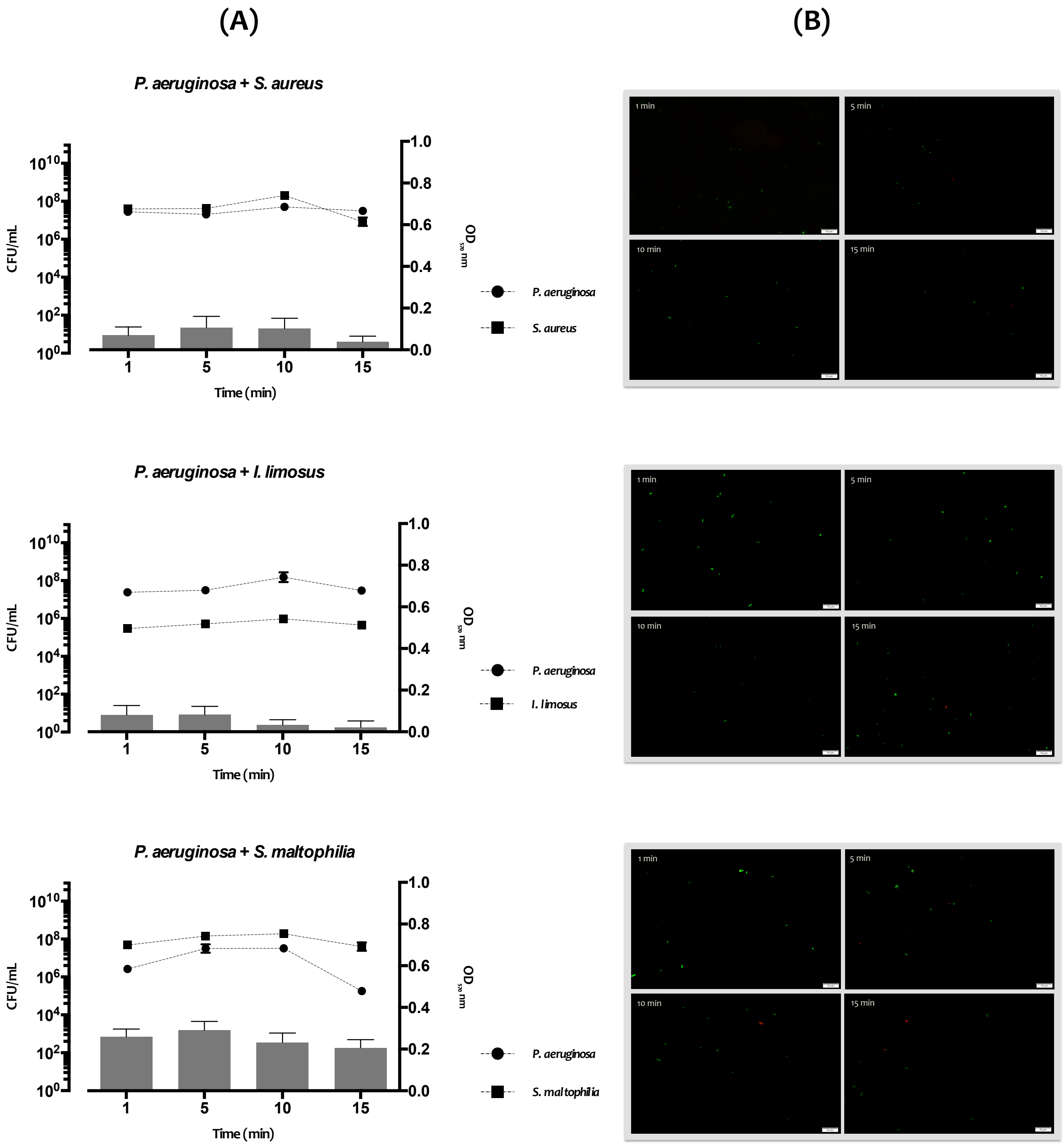

Supplementary Figure 2. Time optimization for 24 h-old biofilm cell detachment in co-cultures of *P.* aeruginosa+S. *aureus, P.* aeruginosa+*I. limosus* and *P.* aeruginosa+*S. maltophilia* under aerobic conditions. **(A)** Biomass (gray bars) and culturable cells (black line) and **(B)** cell viability using SYTO BC/PI (ThermoFisher Scientific) as a LIVE/ DEAD cell viability kit.
